# Willingness to pay for a COVID-19 vaccine and its associated determinants in Iran

**DOI:** 10.3389/fpubh.2023.1036110

**Published:** 2023-02-16

**Authors:** Moslem Soofi, Gerjo Kok, Shahin Soltani, Ali Kazemi-Karyani, Farid Najafi, Behzad Karamimatin

**Affiliations:** ^1^Social Development and Health Promotion Research Center, Health Institute, Kermanshah University of Medical Sciences, Kermanshah, Iran; ^2^Work and Social Psychology Department, Maastricht University, Maastricht, Netherlands; ^3^Research Center for Environmental Determinants of Health, Health Institute, Kermanshah University of Medical Sciences, Kermanshah, Iran

**Keywords:** willingness to pay, COVID-19 vaccine, contingent valuation method, Iran, valuation in health, vaccine preferences

## Abstract

**Introduction:**

Understanding the individuals' willingness to pay (WTP) for the COVID-19 vaccine could help design policy interventions to control the COVID-19 pandemic. This study aimed to estimate the individuals' willingness to pay (WTP) for a COVID-19 vaccine and to identify its associated determinants.

**Methods:**

A cross-sectional survey was conducted on 526 Iranian adults using a web-based questionnaire. A double-bounded contingent valuation approach was used to estimate WTP for the COVID-19 vaccine. The parameters of the model were estimated based on the maximum likelihood method.

**Results:**

A considerable proportion of participants (90.87%) were willing to pay for a COVID-19 vaccine. Based on our discrete choice model, the estimated mean WTP for a COVID-19 vaccine was US$ 60.13 (CI: 56.80–63.46; *p* < 0.01). Having a higher perceived risk of being contaminated with COVID-19, higher average monthly income, higher education level, pre-existence of chronic diseases, previous experience of vaccination, and belonging to higher age groups were significant determinants associated with WTP for COVID-19 vaccination.

**Conclusion:**

The present study indicates a relatively high WTP and acceptance of a COVID-19 vaccine among the Iranian population. Average monthly income, risk perception, education level, the preexistence of chronic disease, and previous vaccination experience increased the likelihood of WTP for a vaccine. Subsidizing the COVID-19 vaccine for the low-income population and raising risk perception among the population should be considered in formulating vaccine-related interventions.

## Introduction

The World Health Organization (WHO) announced COVID-19 to be a pandemic on March 11, 2020 (1). According to WHO (2021), until 14 April 2022, 500,186,525 cases of COVID-19 were confirmed globally, resulting in 6,190,349 deaths. As of November 19, 2021, there have been ~7,199,861 confirmed cases of COVID-19 in Iran, with 140,716 deaths (2). COVID-19 has caused disruptions in multiple sectors of human life and potentially could cause persistent symptoms (3, 4).

Hand-washing, mask-wearing, and social distancing are the primary methods for preventing the spread of COVID-19 in most countries. However, their effectiveness is limited, and they have psychological, economic, and societal impacts on people (5). Vaccination is now the most effective way to protect individuals from communicable diseases and provide long-term immunity (6, 7).

In providing an essential health service (e.g., vaccination) to the people, the evaluation of this service by them is one of the most important issues that can be used to measure their acceptance and the success of the program. The question is how much individuals or society as a whole value the COVID-19 vaccine (8). Willingness to Pay (WTP) is the maximum amount of money that a person would be willing to pay for a specific intervention or service. Several studies on the willingness to pay (WTP) for the COVID-19 vaccine have been conducted in various countries worldwide, including Kenya, Malaysia, Ecuador, and Indonesia (9–12). However, gaps in knowledge regarding WTP for COVID-19 vaccination still exist, especially in low- and middle-income countries. Previous studies have shown that a majority of people in some LMICs are still hesitant to get vaccinated against COVID-19, particularly in the Middle East and North Africa (13).

WTP estimation studies enable decision-makers to have a better understanding of individuals' preferences used in designing health policies (14). Information on individuals' WTP also can be helpful in formulating well-designed health interventions for use in target populations (10). Moreover, pharmaceutical companies can use information regarding individuals' WTP for a COVID-12 vaccine to identify a potential vaccine market and pricing strategy (15). In addition, the social valuation of a vaccine is important to assess the benefits of public and private investments required for its development and distribution.

Until April 14, 2022, ~65 % of people around the world had received at least one dose of the COVID-19 vaccine; however, only 15.2 % of people in low-income countries had received at least one dose (16). While the government has provided free access to COVID-19 vaccines, the vaccine manufacturers must be compensated for their costs related to research and development, production and distribution (8). Although the COVID-19 vaccine is available to individuals free of charge in Iran, like in most countries, estimating the individuals' willingness to pay can indicate their propensity for vaccine-related decisions as well as their understanding of the need for vaccination. Thus, the findings of our study can help health planners gain a better understanding of vaccine-related behaviors and be used as a criterion to compensate vaccine developers in developing countries like Iran. Given that many countries are currently involved in COVID-19 and may face another pandemic in the future, estimating the willingness to pay for this pandemic can highlight the challenges of other possible future pandemics and help design control interventions. The goal of this study is to elicit individuals' WTP for a COVID-19 vaccine and its associated determinants in Iran. Our findings may be useful in formulating public health policies on the COVID-19 vaccine.

## Methods

### Study design and sample

A cross-sectional survey among the Iranian adult population aged 18 and above was conducted. Due to the difficulties of conducting a face-to-face survey at the time of the COVID-19 pandemic, the method of an online survey was used. To determine sample size, we used single population proportion formula by considering a 95% level of confidence, a 5% sampling error or precision limit, and a proportion of individuals who are willing to pay for the COVID-19 vaccine 50%.

The minimum sample size was determined to be 385. However, in order to reduce sample error, the final sample size was increased (*N* = 526). Individuals participated voluntarily through convenience sampling. We sent the web-based questionnaire to individuals via Telegram and WhatsApp. They were requested to forward the questionnaire link to their contacts. We informed participants that their participation in the study is entirely voluntary; thus, they are free to leave at any time, and their completion of the questionnaire represents their informed consent to participate in the study. WhatsApp and Telegram were chosen because these social media platforms are used by the majority of Iranians across socioeconomic groups. As a result, we were able to access the general population both from low and high socioeconomic groups, which is important for WTP studies.

### Survey instrument

The survey instrument was developed by the research team including public health professionals (e.g., a health economist, a social psychologist, etc.). The study tool consisted of a set of binary questions developed to estimate WTP. It also consisted of questions on sociodemographic factors, the perceived risk of being infected with COVID-19, having existing chronic diseases, self-rated overall health status, and COVID-19 vaccination intention. Sociodemographic factors included age, sex, marital status, average monthly income, level of education, and living area (urban/ rural). The average monthly income was categorized as: < IRR 30 million (< US$ 714.28); IRR 30–60 million (US$ 714.28–US$ 1,428.57); and more than IRR 60 million (>US$ 1,428.57). The questions that were used for measuring intention to uptake the COVID-19 vaccine and perceived risk were: “If a safe and effective vaccine against COVID-19 is available, how likely are you to get that?” and “How do you rate your chances of getting COVID-19?”, respectively. The questionnaire was validated qualitatively using expert opinion. In addition, a pilot survey of 50 participants was conducted to identify problematic questions, assess the bid values' validity, and finalize the instrument.

### Econometric estimation and eliciting WTP

To elicit WTP, the contingent valuation approach in its double-bounded dichotomous choice format was used. It allows efficient use of participants' information. It is considered an appropriate method since it is statistically more efficient in estimating the variance of the parameter, resulting in a more precise confidence interval (17–20).

To elicit participants' WTP, they were first asked if they were willing to pay an initial price for a COVID-19 vaccine. If the respondent answered no (yes) to the initial price, they were asked if they were willing to pay a lower (higher) price for the vaccine. Particularly, respondents were asked, “Are you willing to pay 1.5 million Iranian Rials (IRR) (equivalent to US$ 35.71 using an exchange rate of 1 US$ = IRR 42,000) for the COVID-19 vaccine?”. If the respondent replied “yes” to this bid, then, the initial price was doubled to IRR 3 million (US$ 71.43) and if the respondent replied “no” to this bid, then, the initial price was halved to IDR 750,000 (US$ 17.85). In this way, two answers were recorded for each individual. Based on the responses to double-bounded dichotomous questions, the WTP can be estimated in four types of possible intervals:

- “yes-yes”, where, US$ 71.43 ≤ WTP < ∞,
- “yes-no”, where, US$ 35.71 ≤ WTP < US$ 71.43,
- “no-yes”, where, US$ 17.85 ≤ WTP < US$ 35.71, and
- “no-no”, where, 0 ≤ WTP < US$ 17.85

The econometric estimation assumes that WTP can be modeled as follow:


(1) $$\begin{array}{l} WTP_{\text{i}}\left(z_{\text{i}},u_{\text{i}}\right)=z_{\text{i}}^{{}^{\prime }}\beta +u_{\text{i}}\;and\;u_{\text{i}}\approx N\left(0\;,\sigma^{2}\right) \end{array}$$

where z_i_, β and u_i_ represent a vector of explanatory variables, represent a vector of parameters, and an error term, respectively.

The following function was maximized to estimate the parameters of the model:


(2) $$\begin{array}{l} \sum_{i=1}^{N}[d_{i}^{yn}\ln\left(\Phi \left({z^{\prime }}_{i}\frac{\beta }{\sigma }-\frac{71.43}{\sigma }\right)-\Phi \left({z^{\prime }}_{i}\frac{\beta }{\sigma }-\frac{35.71}{\sigma }\right)\right)\\ +\;d_{i}^{yy}\ln\left(\Phi \left({z^{\prime }}_{i}\frac{\beta }{\sigma }-\frac{71.43}{\sigma }\right)\right)+d_{i}^{ny}\ln(\Phi \left({z^{\prime }}_{i}\frac{\beta }{\sigma }-\frac{35.71}{\sigma }\right)\\ -\Phi \left({z^{\prime }}_{i}\frac{\beta }{\sigma }-\frac{17.85}{\sigma }\right))+d_{i}^{nn}\ln\left(1-\Phi \left({z^{\prime }}_{i}\frac{\beta }{\sigma }-\frac{17.85}{\sigma }\right)\right)] \end{array}$$

Furthermore, $d_{i}^{yn}$, $d_{i}^{yy}$, $d_{i}^{ny}$, and $d_{i}^{nn}$ are indicator variables that take the value of one or zero, depending on the relevant case for each individual, implying that a given individual contributes the likelihood function's logarithm in only one of its four parts. Following Lopez-Feldman β and σ were estimated using the maximum likelihood method (21).

## Results

### Descriptive results

Data from 526 individuals were analyzed. The mean age of respondents was 39.11 (SD±11.63). More than half (55.32%) of the respondents were men, 42.78% had a postgraduate degree, 29.28% belonged to the age group of 30-39 and most of the participants (87.07) lived in urban areas. 12.36% of respondents stated that they had previous vaccination experience prior to the pandemic, 59.70 % had a high level of intention to uptake the COVID-19 vaccine and 23.57 of participants had a low level of risk perception (Table 1).

**Table 1:** Characteristics of the participants, cross-sectional survey, 2021.

**Variables**	***N* (%)**
**Sex**	
Male	291 (55.32)
Female	235 (44.68)
**Age group**	
18–29 years	124 (23.57)
30–39 years	154 (29.28)
40–49 years	142 (27.00)
50 years or more	106 (20.15)
**Marital status**	
Married	345 (65.59)
Single/Widowed/Divorced	181 (34.41)
**Education level**	
High school or less	87 (16.54)
Bachelor degree	214 (40.68)
Postgraduate degree	225 (42.78)
**Place of residence**	
Urban	458 (87.07)
Rural	68 (12.93)
**Average monthly income (Million IRR** ^a^ **)**	
< 30	88 (16.73)
30–60	165 (31.37)
>60	273 (51.90)
**Self-rated general health**	
Poor/ Fair	127 (24.14)
Good	210 (39.92)
Very good	189 (35.93)
**Pre-existing condition-respondent**	
Yes	42 (7.98)
No	484 (92.02)
**Level of risk perception**	
Low	124 (23.57)
Moderate	186 (35.36)
High	216 (41.06)
**Having previous vaccination experience**	
Yes	65 (12.36)
No	461 (87.64)
**Level of intention to uptake COVID-19 vaccine**	
High	314 (59.70)
Moderate	100 (19.01)
Low	112 (21.29)

^a^US$ = 42,000 Iranian Rials (IRR).

**Figure 1:**
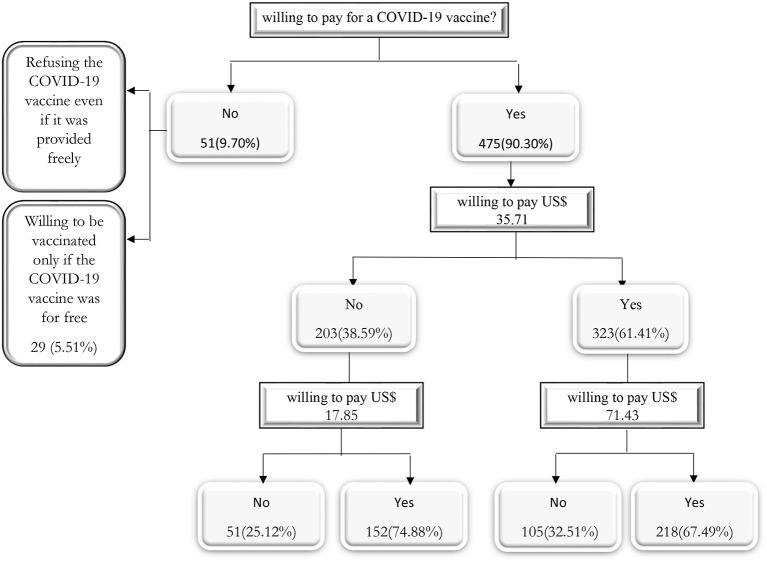
Summary of statistics of the responses to the double-bonded dichotomous choice questions.

Of the sample, 378 (71.86%) participants were willing to pay for a COVID-19 vaccine. The remaining 148 (28.14%) individuals stated that they were unwilling to pay for a COVID-19 vaccine, of which 79 (15.02%) individuals stated that they would refuse the COVID-19 vaccine even if it was given to them for free, and 69 (13.12 %) individuals stated they would only be vaccinated if the vaccine was free. In addition, 218 (41.44 %) of participants stated that they would pay both the initial bid and the higher second bid, while 105 (19.96 %) participants stated that they would pay the initial bid but not the higher second bid (Figure 1).

### Analytical results

The results show that higher educational level, higher income, belonging to older age groups, higher level of risk perception, and having previous vaccination experience are all statistically associated with a higher probability of willingness to pay for a COVID-19 vaccine (Table 2).

**Table 2:** The effect of explanatory variables on the WTP of individuals for COVID19 vaccine.

**Variables**	**Coefficient**	**Standard. Error**	***z***	***P*-value**
**Sex**				
Male	0.41621	3.31732	0.13	0.9
**Age group**				
30–39 years	−4.0312	4.52596	−0.89	0.373
40–49 years	13.8375	5.15931	2.68	0.007
50 years or more	13.8995	5.78919	2.4	0.016
**Marital status**				
Married	−0.4929	3.6696	−0.13	0.893
**Education level**				
Bachelor degree	−0.0221	4.88624	0	0.996
Postgraduate degree	13.5354	5.28502	2.56	0.01
**Place of residence**				
Urban	3.72871	4.88291	0.76	0.445
**Average monthly income (Million IRRa)**				
30–60	5.48579	4.75045	1.15	0.248
>60	19.8117	5.08157	3.9	0.000
**Self-rated general health**				
Good	8.29659	4.11755	2.01	0.044
Very good	15.9019	4.38084	3.63	0.000
**Pre-existing condition**				
Yes	19.0934	6.83717	2.79	0.005
**Level of risk perception**				
Moderate	7.35458	4.28919	1.71	0.086
High	15.646	4.37045	3.58	0.000
**Having previous vaccination experience**				
Yes	16.4942	5.48025	3.01	0.003
_cons	12.2261	7.19541	1.7	0.089
**Sigma**				
_cons	32.1637	1.64028	19.61	0

Sample: 526, Log likelihood = −642.85, Wald chi2(16) = 168.36, Prob > chi^2^ = 0.0000.

Table 3 shows the estimated mean WTP for the basic and expanded model. The basic model is based on the assumption that there are no control variable variables. The expanded model depicted the best-fit model when control variables were taken into account. The results indicated that the mean of the WTP for the COVID-19 vaccine for the basic and expanded model was 60.08 US$ (CI 95%: 56.13–64.04) and 60.13 US$(CI 95%: 56.80–63.46), respectively. The estimated WTP was statistically significant in both models.

**Table 3:** Estimation of double-bounded discrete choice models and willingness-to-pay (WTP) estimates for the basic and expanded model^a^, 2021.

	**Mean WTP ($)**	**Standard. Error**	***P*-value**	**Confidence interval 95%**
Basic model	60.08	2.01	0.000	56.13–64.04
Expanded model	60.13	1.70	0.000	56.80–63.46

^a^Values are in US dollars.

## Discussion

In this study, we estimated the WTP for a hypothetical COVID-19 vaccine and examined its associated determinants in Iran. To our knowledge, this is the first study that estimated the WTP for a COVID-19 vaccine using the contingent valuation method in Iran. We found that most of the respondents (95.81%) were willing to accept a COVID-19 vaccine. These acceptability estimates are similar to those from other countries including Kenya (9), Ecuador (11), Malaysia (12), and Indonesia (10) which reported vaccine acceptability rates >90%. The estimated acceptance rate is higher than those reported from other countries such as Saudi Arabia and Turkey, which reported acceptability rates of ~64 and 80%, respectively (22, 23). In addition, our study demonstrated that the majority (90.30%) of respondents were willing to pay for a COVID-19 vaccine. This finding is higher than those reported in Indonesia (10), Bangladesh (24), Chile (25), and Ecuador (11). Based on our double-bounded dichotomous model, it was estimated that the participants' WTP is US$ 60.1. This finding is comparable in magnitude to those found by studies conducted in Indonesia (10) and Kenya (9). In a study by Carlos E. Carpio et al. in Kenya individuals' mean WTP for the vaccine was estimated to be ranged from USD 49.81 to USD 68.25 (9). On the other hand, the estimated mean WTP for Iran is larger than those found in other studies (12, 24, 26). For example, the estimated mean WTP in our study is about 2 times and 3 times higher than those found in the Malaysian and Brazilian studies that reported a mean WTP of US$ 30.70 and US$ 22.18, respectively (12, 14). However, the estimated mean WTP in our study is low when compared to that of previous studies conducted in Chile, Ecuador, and the US (8, 11, 25, 27). These differences in WTP values could be attributed to differences in the methods used, each country's economic, health, and cultural conditions, and the status of the COVID-19 pandemic at the time of data collection. We estimated individuals' WTP for a COVID-19 vaccine in Iran during the early stages of the disease, and it is now expected to be higher than the amount estimated in this study due to an increase in the number of infected cases, a higher contagion rate, the rapid spread of the disease, global involvement, and a reduction in economic activity (25). Moreover, some of the other influencing factors such as national vaccination policy, vaccine critics' voices, health education messages, severe health consequences of the disease, concerns over adverse effects, etc. had not yet reached their peak impact. These factors can also affect the willingness to pay for the vaccine.

Individuals with a higher risk perception were more likely to pay for a COVID-19 vaccine, according to our findings. Similarly, previous research found that if a person perceived high risk of contracting the disease, she/he would be more willing to pay for the vaccine (10, 28, 29). In Indonesia, for example, having a higher perceived risk has been associated with higher WTP for the COVID-19 vaccine (10). Another study in Vietnam found that low-risk individuals paid less than those who rated themselves as moderate- or high-risk (29). The level of risk perception should be given more attention in public health policy because it is among the most important modifiable determinants of WTP (10). Because this is an important determinant of vaccine acceptance, a well-designed intervention to raise individuals' perceived risk for COVID-19 is required to increase a positive attitude toward vaccination (30). As a result, it is crucial to improve people's awareness well about the COVID-19 pandemic and the importance of the vaccine (29). It should be noted that at this time that the pandemic has passed several peaks in terms of morbidity, mortality and etc., it is expected that the risk perception of individuals should be affected and there may be a higher level of risk perception among the population (25).

According to our findings, participants with a higher average monthly income were more likely to pay for a COVID-19 vaccine. This is consistent with previous studies that found a significant relationship between income and willingness to pay for a COVID-19 vaccine (8, 12, 25). A positive association between income and WTP is used as a justification to subsidize vaccination for the poorest groups. Subsidizing the cost of the COVID-19 vaccine may increase vaccine uptake in terms of public health policy, while higher-income groups may be able to pay for it (9, 25).

Our findings show that high-educated individuals are more likely to pay for the COVID-19 vaccine. This finding is in line with the previous studies that found education as a significant determinant of WTP for the COVID-19 vaccine (12, 25). For example, Wong et al. found that higher education levels resulted in a higher WTP for the COVID-19 vaccine. This finding suggests that vaccination campaigns should target people with a low educational level, as they are less likely to intend to receive a vaccine. A possible explanation is that their health literacy and education levels are likely to be lower, and they may be unaware of the risk of the disease (31).

We have not found a significant association between sex and place of residence with WTP for the covid-19 vaccine, while some studies have reported a significant relationship between these factors and WTP. It should be noted that WTP is influenced by a number of factors, including sociodemographic features and individuals' attitudes and beliefs. These characteristics are not always related to WTP in the same way in different communities (32).

The pre-existing condition also was an important factor that had a positive significant association with WTP for the COVID-19 vaccine. This is consistent with the finding of studies conducted in Chile and the USA (8, 27). One possible explanation is that people with chronic illness have a higher perceived risk due to their condition and health education programs. In addition, in public health policies, these people have been given priority in receiving the vaccine (33). So, these may improve their risk perception and their understanding of the need for vaccines.

Another interesting result from our study is that having previous experiences with vaccination was positively and statistically significantly associated with WTP for the COVID-19 vaccine. A possible explanation for this might be that people who have been vaccinated before have lost some of their fear of the adverse effects of the vaccine.

An important finding was that ~10% of those willing to be vaccinated stated that they were unwilling to pay for the COVID-19 vaccine. Of which, near to half would refuse the COVID-19 vaccine even if it was provided for free. The remaining half stated that they were willing to be vaccinated only if the vaccine was provided freely. Therefore, any policy interventions that are designed should consider these groups as well (25).

There are some limitations to this study. First, we used an online survey as well as convenience sampling to collect the data. This may result in sampling bias, thus, results may not be fully representative of the Iranian population. Second, we conducted this study before the COVID-19 vaccine became available in Iran with a hypothetical vaccine. Therefore, in practice, findings may now differ. In addition, periodic vaccinations may be required, which in turn will affect the willingness to pay. However, we believe that our study has provided some essential evidence concerning the WTP for a COVID-19 vaccine.

## Conclusion

Having higher perceived risk, higher average monthly income, and having characteristics that lead to greater risk perception of developing COVID-19, such as a higher education level, the pre-existence of chronic diseases, etc., are positively associated with WTP. These variables could be considered in designing public health policies to fight against the COVID-19 pandemic. For example, subsidizing the COVID-19 vaccine for the low-income population and raising risk perception among the population should be considered in formulating vaccine-related interventions.

## Data availability statement

The raw data supporting the conclusions of this article will be made available by the authors, without undue reservation.

## Ethics statement

This study was approved by the Ethics Committee of Kermanshah University of Medical Sciences (KUMS). (Ethics code: IR.KUMS.REC.1399.047). Informed consent was obtained from all respondents.

## Author contributions

Conceptualization and methodology: MS and BK. Data curation, formal analysis, funding acquisition, and writing original draft: MS. Project administration: BK. Visualization: BK and FN. Writing review and editing: MS, BK, GK, FN, SS, and AKK. All authors contributed to the article and approved the submitted version.
